# Organoid models: reshaping the paradigm for precision development and evaluation of CAR-T cell therapies

**DOI:** 10.3389/fbioe.2026.1797270

**Published:** 2026-04-15

**Authors:** Haipeng Li, Jiaqi Yan, Yuxi Liu, Xunqian Tao, Keying Guo, Jing Zhang

**Affiliations:** 1 Deparmtent of Mental Health, Bengbu Medical University, Bengbu, Anhui, China; 2 The First Clinical Medical College of Bengbu Medical University, Clinical Medicine Major, Bengbu, Anhui, China; 3 Deparmtent of Stomatology, Bengbu Medical University, Bengbu, Anhui, China; 4 College of Nursing, Bengbu Medical University, Bengbu, Anhui, China

**Keywords:** chimeric antigen receptor T-cell therapy, organoid-on-a-chip, patient-derived tumor organoids, tumor-immune co-culture, tumor-immune microenvironment

## Abstract

Chimeric antigen receptor T (CAR-T) cell therapy has achieved transformative success in hematological malignancies; however, its translation to solid tumors remains severely limited by tumor heterogeneity, immunosuppressive microenvironments, and safety concerns such as on-target/off-tumor toxicity. A major contributor to these challenges is the lack of preclinical models capable of faithfully recapitulating human tumor architecture and tumor-immune interactions. Conventional two-dimensional cell cultures and animal models frequently fail to predict CAR-T efficacy, resistance, and toxicity observed in patients. Organoid technology, particularly patient-derived organoids (PDOs) and immune-integrated organoid systems, has emerged as a next-generation platform that bridges this translational gap. By preserving patient-specific genetic, phenotypic, and spatial heterogeneity, organoids provide a physiologically relevant and scalable system for interrogating CAR-T cell behavior in human-like tumor contexts. Recent advances in tumor-immune co-culture, vascularized organoids, and microfluidic organoid-on-a-chip platforms have further expanded their utility for dynamic assessment of CAR-T infiltration, cytotoxicity, cytokine release, and adaptive resistance mechanisms. In this review, we comprehensively examine how organoid-based models are reshaping the CAR-T development pipeline, spanning target discovery and validation, functional efficacy assessment, safety profiling, and optimization of combination therapies. We further discuss emerging applications of organoids as patient-specific “avatars” for personalized CAR-T selection and response prediction. Finally, we highlight current technical limitations and future bioengineering directions required to enable clinical translation. Collectively, organoid platforms represent a transformative tool for accelerating precision development of next-generation CAR-T cell therapies and advancing human-relevant immuno-oncology research.

## 1 Introduction

### 1.1 Transformative achievements and persistent barriers of CAR-T cell therapy

Chimeric antigen receptor T (CAR-T) cell therapy has fundamentally altered the therapeutic landscape of hematological malignancies, representing one of the most successful clinical translations of synthetic biology and immunoengineering. By genetically redirecting autologous T cells toward tumor-associated antigens in a major histocompatibility complex-independent manner, CAR-T therapies have achieved remarkable complete response rates in B-cell acute lymphoblastic leukemia, diffuse large B-cell lymphoma, and multiple myeloma (Gross et al., 1989). These successes have validated CAR-T cells as a living drug capable of durable tumor control through *in vivo* expansion and long-term persistence.

Despite these advances, the extension of CAR-T therapy to solid tumors has proven substantially more complex and remains largely unsuccessful in the clinic. Unlike hematologic malignancies, solid tumors are characterized by profound spatial and molecular heterogeneity, a dense stromal architecture, and a highly immunosuppressive tumor microenvironment (TME) (Zhu et al., 2024; Kumar et al., 2024; Montironi et al., 2021). Barriers to effective CAR-T therapy include limited trafficking and infiltration into tumor tissue, antigen heterogeneity and loss leading to immune escape, chronic T-cell exhaustion driven by persistent antigen exposure, and potent immunosuppressive signals mediated by regulatory T cells, myeloid-derived suppressor cells, tumor-associated macrophages, and inhibitory cytokines (Kalaitsidou et al., 2015; Tomai et al., 2025).

Equally concerning are safety-related challenges. On-target/off-tumor toxicity remains a major risk when target antigens are expressed at low levels in essential normal tissues, while excessive CAR-T activation can precipitate cytokine release syndrome and immune effector cell-associated neurotoxicity (Lee et al., 2014; Lamers et al., 2016; Morris et al., 2022). Collectively, these obstacles underscore the urgent need for improved preclinical systems capable of capturing the complexity of human solid tumors and accurately predicting CAR-T therapeutic performance and risk.

### 1.2 The preclinical modeling bottleneck in CAR-T development

The translational failure of many CAR-T strategies in solid tumors is increasingly recognized as a consequence of inadequate preclinical modeling. Conventional two-dimensional cancer cell lines, while experimentally tractable, lack three-dimensional architecture, fail to recapitulate intratumoral heterogeneity, and do not model physiologically relevant gradients of oxygen, nutrients, and metabolites (Kapałczyńska et al., 2018; Edmondson et al., 2014). As a result, CAR-T cytotoxicity assessed in 2D cultures often overestimates therapeutic potency and fails to predict resistance mechanisms observed in patients.

As mentioned earlier, animal models, particularly patient-derived xenografts (PDXs), while exhibiting greater structural complexity, still possess significant limitations (Hidalgo et al., 2014; Meraz et al., 2019; Schneeberger et al., 2016). Most CAR-T studies rely on immunodeficient mouse hosts, which preclude evaluation of endogenous immune regulation and toxicity. Even humanized mouse models incompletely recapitulate human immune dynamics and are associated with high cost, long generation times, and limited scalability (Shultz et al., 2012; Laudanski et al., 2018; Daharsh et al., 2019).

This disconnect between preclinical models and clinical reality has created a translational bottleneck in CAR-T development, contributing to late-stage failures and unpredictable toxicity profiles. Consequently, there is a growing consensus that next-generation human-relevant models are required to de-risk CAR-T therapies earlier in the development pipeline.

### 1.3 Emergence of organoid technology as a transformative tumor modeling platform

Organoid technology has rapidly emerged as a powerful solution to this challenge. Derived from adult stem cells, pluripotent stem cells, or primary tumor tissue, organoids self-organize into three-dimensional structures that recapitulate key architectural, genetic, and functional features of native tissues. Tumor-derived organoids, particularly patient-derived organoids (PDOs), retain the histopathological characteristics, genomic alterations, transcriptional programs, and phenotypic heterogeneity of the parental tumor across extended culture periods.

Importantly, organoids occupy a unique position between reductionist *in vitro* systems and complex *in vivo* models. They offer human specificity, experimental accessibility, and scalability while maintaining a degree of biological complexity unattainable in 2D cultures. These properties have positioned organoids as a cornerstone technology for disease modeling, drug discovery, and increasingly, immunotherapy research.

Recent advances have further expanded organoid utility through integration with immune cells, stromal components, vascular networks, and microfluidic systems. These innovations have enabled direct interrogation of tumor-immune interactions under controlled yet physiologically relevant conditions, making organoids particularly attractive for CAR-T cell research.

### 1.4 Scope and objectives of this review

**FIGURE 1:**
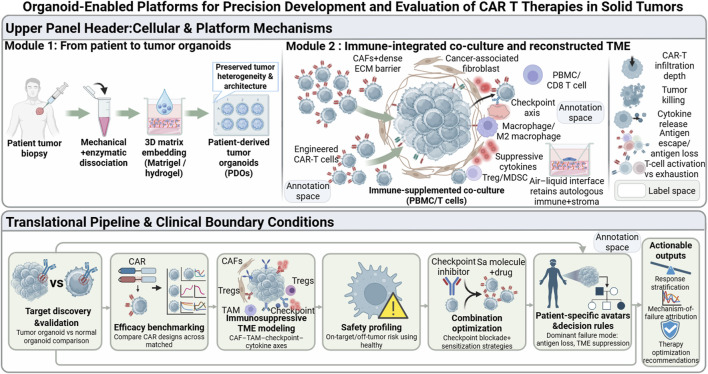
Organoid-Enabled Platforms for Precision Development and Evaluation of CAR-T Therapies in Solid Tumors. Part A demonstrates the construction and immunological co-culture analysis of solid tumor organoids. Patient tumor tissues were dissociated and embedded in matrix gel to generate patient-derived tumor organoids that preserve intratumoral heterogeneity. Subsequently, an immunosuppressive tumor microenvironment was constructed by integrating peripheral blood mononuclear cells (PBMCs), enriching CD8^+^ T cells and key inhibitory components (e.g., cancer-associated fibroblasts, M2-polarized macrophages, regulatory T cells), or utilizing air-liquid interface culture to preserve autologous immune matrix components. This system enables systematic evaluation of CAR-T cell infiltration depth, killing kinetics, cytokine secretion, antigen escape, and the dynamic trajectory from T cell activation to exhaustion. Part B presents an organoid-guided translational research workflow. This process encompasses: (1) Target discovery and validation (tumor vs. normal organoid comparisons); (2) Standardized testing of CAR structure and manufacturing strategies; (3) Modeling of immunosuppressive microenvironments (integrating CAFs, Tregs, and checkpoint signaling axes) to decipher resistance mechanisms; (4) Safety evaluation (using healthy tissue organoids or multi-organ integrated systems); (5) Optimization of combination therapy regimens (combined with checkpoint inhibitors or small-molecule drugs). Ultimately, integrating these data constructs patient-specific organoid virtual models to identify key failure mechanisms, perform response stratification and mechanism attribution, and provide decision-making support for clinical treatment optimization.

In this review, we provide a comprehensive and forward-looking analysis of how organoid technologies are reshaping the development, evaluation, and personalization of CAR-T cell therapies. We first summarize foundational principles and recent advances in tumor organoid engineering. We then systematically examine organoid-enabled applications across the CAR-T development continuum (Figure 1), including antigen discovery, functional efficacy assessment, toxicity prediction, and optimization of combination strategies. Finally, we discuss the emerging role of organoids as patient-specific “avatars” for precision immunotherapy, critically evaluate current limitations, and outline future directions at the intersection of bioengineering, immunology, and translational medicine.

## 2 Organoid technology: from foundational principles to advanced tumor models

### 2.1 Methodological foundations of tumor organoid generation

Organoids can be generated directly from patient tumor tissue or from stem cell sources, and the major organoid model formats used across CAR-T research are summarized in Table 1. For patient derived tumor organoids, freshly obtained specimens are typically subjected to mechanical dissociation and enzymatic digestion to produce single cells or small tissue fragments, which are then embedded in a three dimensional matrix and cultured in a defined medium containing tumor type specific growth factors and signaling pathway modulators. Media are commonly optimized by adding Wnt3a, R spondin, epidermal growth factor (EGF), and the bone morphogenetic protein antagonist Noggin to sustain stem cell self renewal and enable the emergence of organ like structures (Zhang et al., 2020; Wilson et al., 2020). With this standard submerged Matrigel culture approach, organoids can be established across many solid tumors, with overall success rates exceeding 50% in most tumor types and reaching approximately 80% in selected cancers such as colorectal, breast, and ovarian carcinoma (Sachs et al., 2018; Veninga and Voest, 2021). Mechanistically, preservation of native tissue architecture can drive fidelity and yield, as Jacob et al. reported that directly culturing microdissected glioblastoma fragments helps maintain vulnerable cellular subsets and hypoxia gradients, thereby improving establishment success and more faithfully capturing microenvironmental features (Jacob et al., 2020a; Jacob et al., 2020b). Likewise, Fujii et al. showed that some colorectal cancers carrying intrinsic Wnt pathway mutations can proliferate without exogenous R spondin or Wnt3a, indicating that medium composition should be tailored to the tumor’s genetic background (Fujii et al., 2016). Accordingly, organoid derivation requires source informed optimization to maximize both establishment efficiency and model representativeness.

**TABLE 1:** Types of organoid models and their features in CAR-T research.

Model type	Origin	Composition
Patient-Derived Organoid	Patient tumor tissue	Primarily tumor cells; lacks endogenous immune cells; minimal stroma; no vasculature
PSC-derived Organoid	Pluripotent stem cell differentiation	Models native organ architecture; can introduce tumor mutations *via* genetic engineering; typically no immune cells; requires matrix support
Organoid-Immune Co-culture	Tumor organoid + exogenous immune cells	Tumor organoids co-cultured with immune cells to partially reconstitute the immune microenvironment
Organoid + Stromal Integration	Tumor organoid + stromal support cells	Incorporates stromal cells into organoid to form ECM and secretion networks; partially mimics tissue architecture and mechanics
Vascularized Organoid/On-chip	Organoid co-cultured with endothelial cells, or cultured in perfused microfluidic chip	Incorporates microvascular networks or perfusion, providing oxygen/nutrient gradients; partially reconstitutes tumor vasculature

Pluripotent stem cell, PSC, derived organoids provide a complementary route to organoid generation beyond direct derivation from patient tissues. For example, induced pluripotent stem cells, iPSCs, can be guided through *in vitro* directed differentiation to produce organoids of the brain, liver, and intestine for disease modeling and drug research (Lancas et al., 2013; Fox and Duncan, 2013; Spence et al., 2011; Sharma et al., 2020). Driven by genetic engineering, PSC based platforms can be adapted from modeling normal development or inherited disorders to generate tumor like organoid models bearing defined oncogenic mutations, offering a rationale for constructing tumor organoids with controlled genotypes (Bian et al., 2018; Rozanska et al., 2022). Bioengineering strategies are further broadening organoid design space, including microfluidic chips that automate culture while enabling tight microenvironmental control, and 3D bioprinting that spatially programs cell placement to recapitulate tissue microarchitecture. These emerging approaches are expected to improve construction efficiency, reproducibility, and model diversity, thereby supporting the development of more complex *in vitro* systems (Papamichail et al., 2025; Brassard et al., 2021).

### 2.2 Biological fidelity and engineering advantages of organoids

High-fidelity *ex vivo* preservation of primary-tumour biology and heterogeneity underpins the growing value of organoid models. Across multiple studies, patient-derived tumour organoids closely match their paired patient tumours in tissue architecture, genetic and molecular features, and drug-response phenotypes (van de Wetering et al., 2015). Organoids typically recapitulate the histopathological morphology of the index tumour, including glandular or solid growth patterns, while maintaining tumour-cell polarity and spatial organisation (Sachs et al., 2018; Canet-Jourdan et al., 2022). At the genomic level, organoids capture most of the parental tumour’s mutational and transcriptional landscape; tumour organoids have been reported to retain more than 80% of mutations present in the originating tumour and to preserve expression of key oncogenic driver genes (Lee et al., 2018). Mechanistically, the strong transcriptomic concordance between organoids and parental tumours is reflected by high agreement in major signalling programmes and molecular subtype assignment across organoid biobanks (van de Wetering et al., 2015). Functionally, organoids reproduce patient-specific sensitivity or resistance to chemotherapy and targeted agents, and this fidelity is directly relevant to CAR-T development because well-matched organoids can be used *in vitro* to predict tumour susceptibility to CAR-T cells (Mo et al., 2022; Schnalzger et al., 2019).

Another advantage of tumor organoids is their capacity to preserve cellular diversity and the tumor’s subclonal composition. Unlike monoclonal cell lines, organoid cultures can maintain cells with distinct differentiation states and genotypes, thereby approximating intratumoral heterogeneity (Sachs et al., 2018; Broutier et al., 2017; Drost and Clevers, 2018). Consistent with this, a single-cell sequencing study showed that organoids derived from individual cells exhibit diversity in major histocompatibility complex class I (MHC-I) peptide presentation, suggesting that organoids can recapitulate antigen-presentation differences across tumor subpopulations (Demmers et al., 2020). Preserving such heterogeneity is particularly important for evaluating immunotherapies such as CAR-T cells, because intratumoral diversity is a frequent barrier to durable efficacy. By monitoring the survival and interactions of distinct cellular subpopulations within organoids, researchers can more precisely dissect CAR-T cell activity in heterogeneous tumors. Overall, organoid models, owing to their high biological fidelity, provide a more reliable and clinically proximate platform than conventional systems for studying tumor biology and assessing therapeutic responses (van de Wetering et al., 2015; Jin et al., 2018; Ding et al., 2022).

### 2.3 Advanced organoid systems for immunotherapy research

Although conventional tumor organoid models recapitulate many tumor intrinsic features, the absence of immune cells, vasculature, and other essential components prevents them from fully capturing the complexity of the *in vivo* tumor microenvironment, motivating immune-, stromal-, and vasculature-integrated organoid formats (Table 1) (Yip et al., 2023; Bae et al., 2022). To address this gap, a range of next-generation organoid systems has emerged, in which additional cellular and structural elements are incorporated to improve physiological relevance. One representative approach is the immune organoid, where immune populations are introduced into organoid co culture to partially reconstruct the tumor immune milieu (Dijkstra et al., 2018; Baisiwala et al., 2026). For example, Dijkstra and colleagues co cultured patient derived peripheral blood mononuclear cells with colorectal cancer and non small cell lung cancer organoids and observed marked expansion of CD8 positive T cells after 2 weeks (Dijkstra et al., 2018). This finding indicates that exogenous immune supplementation can generate measurable immune infiltration within organoid cultures, providing a tractable platform to interrogate CAR-T cell behavior in a tumor like context (Schnalzger et al., 2019).

However, simply adding immune cells remains insufficient to approximate the full *in vivo* immune ecosystem, because the supplemented repertoire and functionality may be incomplete and the tumor immune interaction window is relatively short (Mahadevan et al., 2023). A complementary strategy is to preserve, as much as possible, the endogenous immune and stromal compartments present within the original tumor tissue. Neal and colleagues developed an air liquid interface culture method that maintains tumor cells together with tumor associated stromal elements, with the basal surface exposed to medium and the apical surface exposed to air (Li et al., 2014). This configuration supports oxygen and nutrient gradient formation that enables sustained organoid growth and enlargement, while allowing resident tumor infiltrating lymphocytes to survive and remain functional; notably, the TILs retained a T cell receptor repertoire comparable to the parental tumor, and exposure to anti PD-1/PD-L1 antibodies activated tumor reactive TILs in a manner consistent with checkpoint blockade responses (Li et al., 2014). By preserving key microenvironmental constituents without exogenous immune addition, this autologous immune organoid better maintains tumor contextual realism and strengthens the credibility of organoid based immunotherapy studies (Yan et al., 2023).

Stromal components beyond immune cells, including fibroblasts, can be incorporated into these models. Cancer associated fibroblasts (CAFs) densify tumour tissue by depositing extracellular matrix and collagen and release multiple immunosuppressive mediators, contributing to limited CAR-T penetration and accelerated exhaustion in solid tumours (Kankeu Fonkoua et al., 2022). Incorporating CAFs into organoid cultures recapitulates the dense stromal barrier of solid tumours, enabling assessment of CAR-T migration and persistence under physical constraint, as well as the extent to which stroma depleting agents restore CAR-T infiltration and effector function (Paterson et al., 2022). Driven by organoid co culture designs that integrate suppressive immune subsets with stromal elements, an immunosuppressive tumour microenvironment can be reconstructed *in vitro* to systematically interrogate the mechanisms by which these cues modulate CAR-T efficacy (Magré et al., 2023). This platform further supports attribution of functional impairment to dominant inhibitory circuits and testing whether combinatorial blockade of these pathways meaningfully enhances CAR-T cytotoxic activity (Xiao et al., 2023). Collectively, such evidence provides a mechanistic basis for rational microenvironment targeted combination regimens and for optimising CAR-T strategies in solid tumours. A structured comparison of these organoid model types, including composition, stage-specific applications, and key advantages and limitations for CAR-T studies, is provided in Table 1.

## 3 Organoid-enabled applications across the CAR-T development pipeline

Among emerging preclinical platforms, organoids are uniquely positioned to interrogate the multidimensional challenges facing CAR-T therapy in solid tumors. Unlike traditional systems that evaluate CAR-T function in isolation, organoid-based models enable simultaneous assessment of tumor heterogeneity, spatial architecture, immune infiltration, and dynamic resistance mechanisms. This section systematically examines how organoids empower CAR-T research at critical stages of development, with representative studies across stages summarized in Table 3.

### 3.1 Target discovery and functional validation in human-relevant contexts

Organoid models can accelerate the discovery and validation of new CAR-T targets: leveraging patient derived tumor organoid biobanks established from multiple individuals, investigators can systematically profile cell surface antigen landscapes to nominate molecules with high suitability as CAR targets (Abrantes et al., 2025). Because organoids largely preserve the antigenic features of the corresponding primary tumors, immunohistochemistry, single cell sequencing, and mass spectrometry based workflows can be applied to compare the prevalence and abundance of candidate antigens across tumor entities and across patients in a standardized manner (Di Paola et al., 2025). In parallel, benchmarking tumor organoids against normal organoids derived from healthy tissues enables early appraisal of antigen expression in non malignant contexts, thereby flagging potential off tumor, on target toxicities before clinical translation (Suadicani et al., 2000). Collectively, organoid biobanks provide a scalable *in vitro* evidence base for CAR target prioritization and may shorten the path to identifying actionable targets for solid tumors.

**FIGURE 2:**
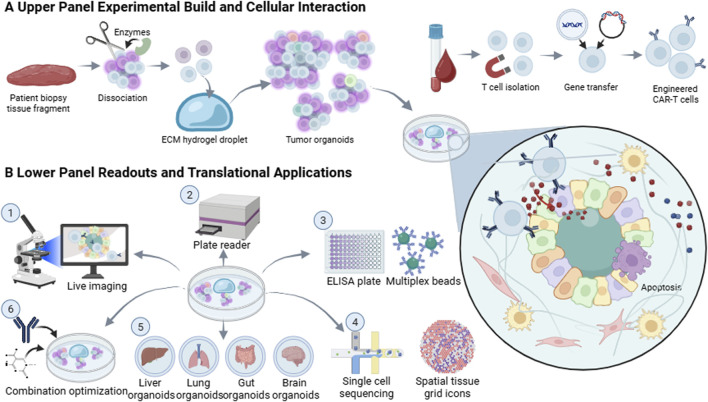
Organoid–CAR-T co culture model. Figure 1. **(A)** illustrates the experimental setup and cell interaction model. Patient tumor tissue is dissociated and embedded in extracellular matrix hydrogels to generate patient-derived tumor organoids. Concurrently, peripheral blood T cells from patients or healthy donors are isolated and genetically modified to obtain CAR-T cells. CAR-T cells are co-cultured with tumor organoids to simulate immune cell infiltration and tumor recognition in a three-dimensional space. An enlarged schematic illustrates the dynamic interactions among CAR-T cells, malignant cells, and tumor microenvironment components (including stromal cells and myeloid cells) within a shared microenvironment, with cytokine diffusion and tumor cell apoptosis serving as representative functional endpoints. **(B)** presents a multimodal detection and translational application system. (1) Live-cell imaging quantitatively analyzes CAR-T infiltration depth and killing kinetics; (2) Multiplex cytokine assays evaluate effector function and inflammatory burden; (3) Single-cell sequencing resolves immune and tumor transcriptional states, tracking activation, exhaustion, and antigen escape pathways; (4) Spatial transcriptomics correlates cellular localization with functional phenotypes; (5) Multi-organ organoid safety assessment (liver, lung, intestine, brain) predicts off-target toxicity; (6) Co-culture systems integrate pharmacologic or immunomodulatory interventions to support combination therapy optimization and patient stratification.

Once candidate targets are nominated, organoid-based co-culture offers a practical, high-fidelity functional readout for CAR-T validation that better captures three-dimensional tumor architecture and microenvironmental constraints than conventional two-dimensional cytotoxicity assays (Jassin et al., 2025a) (Figure 2). Investigators can co-incubate engineered CAR-T cells with tumor organoids expressing the cognate antigen and quantify interaction dynamics and effector outcomes using complementary orthogonal assays (Table 2) (Wang J. et al., 2025). Zou et al. enabled real-time visualization of organoid apoptosis with a Caspase-3/7 activity fluorescent probe, whose signal increased in parallel with CAR-T-mediated killing. Driven by reporter-enabled phenotyping, Schnalzger et al. introduced reporter genes into tumor organoids and measured declines in organoid fluorescence intensity as a quantitative surrogate of CAR-T cytotoxic efficacy (Zou et al., 2021). Yu et al. tracked the intratumoral distribution and kinetics of CAR-T cells by labeling for CD8 and granzyme B. Functional outputs were profiled *via* flow cytometry and immunoassays, measuring lactate dehydrogenase release along with cytokine levels, including interleukin-2, tumor necrosis factor-α, and interferon-γ, in culture supernatants (Schnalzger et al., 2019; Yu et al., 2021).

**TABLE 2:** CAR-T functional evaluation methods using organoid models.

Method	What it measures	Features	Notes
Real-time Imaging	Dynamic tumor cell apoptosis or survival in organoid	Real time monitoring of CAR-T induced organoid cell death by fluorescent probe labeling Enables continuous observation of CAR-T mediated killing of organoids, acquisition of kinetic parameters such as killing rate, and identification of functional differences between highly active and poorly active CAR-T products	Requires live cell imaging microscopy equipment, and the data analysis workload is substantial
Histology and IF Imaging	T cell infiltration into organoid; tumor cell death markers	After co culture, fix organoids, prepare tissue sections or perform whole mount clearing, and conduct immunofluorescence staining Allows assessment of the extent to which CAR-T cells infiltrate into the organoid interior and the degree of tumor cell death Provides spatial distribution information of CAR-T cells within 3D tumors, supporting evaluation of infiltration capacity	Time consuming procedures, difficulty in quantitative analysis, and limited sample throughput
Flow Cytometry	Cell phenotype changesand activation	Digest the organoid–CAR-T co culture system into a single cell suspension, and use flow cytometry to quantitatively analyze CAR-T cell activation or functional states and the proportion of residual tumor cells; Enables multiparameter readouts simultaneously and precise comparison of CAR-T activation levels and killing efficiency under different conditions	Sample preparation requires enzymatic dissociation, which may lead to loss of a fraction of cells Endpoint flow cytometry analysis cannot capture spatial distribution
Cytokine Release Assays	Levels of cytokines	Collect supernatants from organoid–CAR-T co cultures, and analyze CAR-T secreted effector molecules by ELISA or multiplex cytokine assays Cytokine profiles can reflect CAR-T functional strength and potential adverse effects Cytokine measurements are sensitive and efficient	Cannot distinguish which specific cell type produces the factors, and interpretation requires integration with other data
Co-culture Duration &Ratio	Impact of co-culture time and effector: target ratio	Evaluate CAR-T effects at different co culture durations: short term (24–72 h) co culture is used to observe immediate cytotoxic effects Long term (>1–2 weeks) culture can monitor CAR-T persistence and dynamic changes such as exhaustion Adjusting the effector to target cell ratio can influence killing efficiency In culture setup, static suspension co culture can be compared with dynamic flow based co culture, where the former is operationally simple and the latter is closer to physiological conditions but has higher complexity	The study design needs to flexibly configure these conditions according to the specific evaluation focus

Collectively, these organoid assays resolve not only killing magnitude but also killing tempo and CAR-T state transitions, including activation and exhaustion phenotypes. In practice, organoid models have already been used to corroborate the activity of emerging CAR-T constructs; for example, a study targeting a novel hepatocellular carcinoma antigen co-cultured the engineered CAR-T product with patient-derived liver cancer organoids, demonstrating efficient elimination of tumor cells and induction of target-restricted immune responses (Zou et al., 2021). Overall, organoids provide a direct preclinical window for de-risking CAR-T candidates before clinical testing by substantiating tumor-selective recognition and cytotoxicity in a more physiologically grounded context.

### 3.2 Quantitative assessment of CAR-T cell function and killing dynamics

Organoid-based platforms enable multidimensional assessment of CAR-T cell effector function (Table 2), with dynamic imaging providing direct, real-time readouts of CAR-T behaviour within three-dimensional tumour architecture (Figure 2). Organoids offer a tractable system for quantifying infiltration efficiency. Unlike two-dimensional monolayers, organoids incorporate extracellular matrix cues and densely packed cellular aggregates that more closely approximate the physical constraints of solid tumours, thereby permitting evaluation of intratumoural penetration depth and infiltration kinetics (Jassin et al., 2025b). By fluorescently labelling CAR-T cells and applying live-cell microscopy, investigators can track stepwise entry from the organoid periphery to the core and the subsequent recognition and elimination of deeply situated malignant cells (Jassin et al., 2025b). Mechanistically, these three-dimensional imaging analyses delineate how stromal and matrix-derived barriers modulate therapeutic activity and expose CAR design-dependent differences in intratissue motility. In one pancreatic cancer study, CAR-T cells were embedded within the Matrigel layer surrounding patient-derived organoids; the cells progressively infiltrated into the organoid core and established sustained tumour-cell contacts, recapitulating the process by which CAR-T cells overcome matrix barriers to achieve deep infiltration in solid tumours and enabling comparative evaluation of distinct engineering strategies (Fan et al., 2025).

Organoids also permit granular interrogation of CAR-T cell functional states, including activation, proliferation, and the temporal dynamics of exhaustion during tumor cell killing (Jassin et al., 2025a). After CAR-T cells are co cultured with organoids for a defined interval, the recovered cells can be phenotyped by single cell RNA sequencing or flow cytometry to determine whether functional attenuation is emerging. For example, single cell profiling has shown that, relative to the baseline state, organoid exposed CAR-T cells upregulate transcriptional programs enriched for exhaustion associated and memory related genes (Yin et al., 2025), capturing functional state remodeling under sustained tumor engagement. Using exhaustion and persistence readouts as primary endpoints, this platform can test whether interventions such as co stimulatory circuit engineering or cytokine supplementation drive more durable CAR-T activity. In addition, cytokine concentrations and the proportion of residual tumor cells in the co culture provide quantitative efficacy readouts that complement phenotypic analyses (Strijker et al., 2025).

Collectively, integrating dynamic imaging with multi omics in organoid models enables a panoramic assessment of intratumoral CAR-T behavior, including directional infiltration, killing kinetics, duration of effector function, and trajectories of functional decline, thereby providing an evidence base for rational CAR- T optimization.

### 3.3 Modeling immunosuppressive tumor microenvironments

An immunosuppressive tumour microenvironment is a major determinant of limited CAR-T cell efficacy in solid tumours. By incorporating these suppressive components into organoid platforms, one can establish an *in vitro* model of a suppressive tumour microenvironment to interrogate how suppression shapes CAR-T function and to evaluate candidate interventions (Zarychta et al., 2024). Specifically, regulatory T cells (Tregs) and myeloid-derived suppressor cells (MDSCs) suppress CAR-T cells through both contact-dependent mechanisms and the secretion of inhibitory cytokines such as IL-10, TGF-β, and adenosine. Organoid co-culture systems that incorporate defined ratios of these suppressor cells alongside CAR-T cells can faithfully recapitulate these multifaceted suppressive axes (Zhao et al., 2026). For instance, Treg-mediated inhibition of CAR-T activation and proliferation can be quantitatively assessed *via* dynamic imaging of immune synapse formation and cytokine profiling within the three-dimensional organoid architecture—a level of analysis that is largely inaccessible in conventional two-dimensional cultures. Similarly, incorporating monocyte-derived M2-polarized tumour-associated macrophages (TAMs) into tumour organoids recapitulates TAM-mediated suppression through IL-10 and TGF-β signalling, as well as through metabolic competition (Tabe et al., 2025; Yu et al., 2024). This platform enables not only pharmacological testing of interventions such as CSF1R inhibition but also mechanistic dissection of how TAMs compromise CAR-T metabolic fitness and persistence (He et al., 2025). Importantly, these models allow researchers to determine whether CAR-T dysfunction is reversible upon checkpoint blockade or targeted ablation of suppressive populations, providing insights into the dynamics of immune recovery within a human tumour context.

Beyond immune constituents, the tumour vascular network and biophysical constraints within the tumour microenvironment are also major determinants of CAR-T efficacy. Endothelial cells lining the tumour vasculature actively regulate CAR-T cell extravasation through expression of adhesion molecules (e.g., ICAM-1, VCAM-1) and chemokines (e.g., CXCL9, CXCL10), while perivascular cells and the basement membrane impose additional physical barriers. Moreover, aberrant tumour vasculature often exhibits compromised barrier function and heterogeneous perfusion, creating hypoxic and acidic niches that further impair CAR-T cell survival and function. This recognition has driven the emergence of vascularised organoids and organoid-on-chip platforms that more closely approximate *in vivo* conditions. Sontheimer-Phelps et al. developed a microfluidic three-dimensional culture strategy to grow organoids on-chip, recapitulating tissue-level mechanical forces and nutrient gradients. These organoid chips more faithfully reproduce the pathophysiological milieu experienced by tumours *in situ*, while providing an appropriate extracellular-matrix scaffold and haemodynamic environment that preserve native cell-cell and cell-matrix interactions (Kasendra et al., 2018). Within this setting, tumour-immune crosstalk and immune-cell infiltration into the organoid tissue are enhanced (Mattei et al., 2021). In parallel, efforts are being directed toward engineering microvascular structures by integrating endothelial cells into organoids, or coupling organoids with microvascular chips to model tumour perfusion and barrier function (Zhang et al., 2021). Such vascularised models enable quantification of CAR-T entry from the vasculature into tumour tissue and dissection of endothelial and stromal barriers that constrain activity (Dey et al., 2022). Crucially, these systems can reveal whether poor CAR-T infiltration stems from inadequate adhesion, impaired chemotaxis, or physical entrapment—mechanistic questions that are difficult to address with conventional *in vivo* imaging or static cultures. However, current models still face challenges in fully recapitulating the dynamic and heterogeneous nature of the tumour vasculature, such as the intermittent blood flow and pericyte coverage abnormalities seen in patients, highlighting an area for future technical refinement.

Collectively, integrating immune organoids, vascularised organoids, and organoid-on-chip systems offers an unprecedented means to reconstruct the tumour microenvironment and interrogate CAR-T behaviour under physiologically relevant complexity. Unlike traditional platforms, these advanced models capture the spatial organization, multicellular interactions, and dynamic gradients that define the suppressive tumour milieu, thereby enabling mechanistic insights that are both human-specific and clinically actionable. For example, they can distinguish between suppression mediated by direct cell–cell contact *versus* soluble factors, or identify whether CAR-T failure occurs at the level of infiltration, activation, or persistence. As these platforms mature, they will allow finer *in vitro* modelling of the sequential biological events following CAR-T infusion, yielding more predictive evidence to guide therapeutic optimisation. Future iterations should aim to incorporate additional layers of complexity—such as patient-derived stromal components and dynamic immune recruitment—while maintaining experimental tractability, ultimately bridging the gap between preclinical models and patient responses.

### 3.4 Safety profiling and prediction of on-target/off-tumor toxicity

Before clinical deployment of CAR-T therapy, rigorous safety profiling is indispensable. Organoid models offer a distinctive preclinical tool to anticipate off target toxicity and systemic adverse events associated with CAR-T cells. To interrogate on target, off tumor toxicity, organoids derived from healthy tissues can be used to assess CAR-T mediated damage to nonmalignant cells (Schnalzger et al., 2019). When the intended antigen shows low level expression in specific normal tissues, coculturing CAR-T cells with matched healthy tissue organoids enables direct evaluation of normal cell injury and apoptosis (Harter et al., 2024). The reproducible disruption of normal organoids suggests the presence of clinically significant safety risks. Therefore, it is imperative to reassess CAR design or identify more tumor-specific targets at an early stage. Incorporating healthy tissue organoid screening can thus identify and mitigate potential safety hazards before initiating first in human studies (Schnalzger et al., 2019).

Another safety concern is systemic immune toxicity, particularly cytokine release syndrome (CRS). Although fully recapitulating whole-body responses requires complex models, organoid-based co-culture systems can still provide early predictive cues (Meeus et al., 2025). In co-cultures of CAR-T cells with tumor organoids, measuring key cytokines in the culture supernatant offers a first-pass readout of CAR-T activation and the potential intensity of inflammatory responses. If a CAR-T product induces unusually high levels of inflammatory mediators *in vitro*, it may be more likely to provoke severe CRS *in vivo* (Teachey et al., 2016), in which case engineering the CAR-T cells to attenuate excessive cytokine production may be considered (Sterner et al., 2019). In parallel, multi-organ organoid platforms have been developed to more comprehensively evaluate systemic toxicity of drugs and living-cell therapies. For example, Skarda et al. built an “organoid-on-a-chip” system in which cardiac, pulmonary, and hepatic organoids are interconnected on a microfluidic device, enabling simultaneous assessment of drug effects across major organs (Skardal et al., 2017). This concept can be extended to CAR-T safety testing by incorporating tumor organoids together with organ-relevant normal organoids on a similar platform, perfusing CAR-T cells through the circuit, and monitoring injury to normal organoids as well as system-level inflammatory outputs to better approximate *in vivo* conditions. While such complex models are still under active development, ongoing technical advances may position them as an important complementary tool for CAR-T safety evaluation. Overall, organoid-based safety assessment can facilitate early identification and quantification of potential risks, thereby guiding CAR-T design optimization and clinical risk mitigation.

### 3.5 Organoids as platforms for optimizing combination therapies

Organoid platforms can also be leveraged to optimise combination strategies that pair CAR-T therapy with other anticancer modalities (Ning et al., 2024; Qin and Xu, 2022). Driven by an immunosuppressive tumour microenvironment and other layered barriers in solid tumours, CAR-T monotherapy often falls short, making synergistic combinations a major focus of current research (Qin and Xu, 2022). Using organoid co-culture systems, candidate regimens can be screened systematically before clinical testing, enabling evidence-based prioritisation. Typical combinations include CAR-T cells with immune checkpoint inhibitors, as well as CAR-T cells paired with small-molecule targeted agents or cytotoxic chemotherapy (Zhu et al., 2025a). In organoid assays, the addition of anti-PD-1 or anti-CTLA-4 antibodies can model concurrent checkpoint blockade, and readouts such as tumour-cell viability within organoids alongside CAR-T functional metrics can be used to determine whether checkpoint inhibition enhances CAR-T cytotoxicity (Magré et al., 2023). If organoid experiments show that anti-PD-1 co-treatment markedly increases CAR-T infiltration and tumour-cell apoptosis, this supports subsequent validation in animal models and, ultimately, clinical evaluation.

In tumour organoids with high expression of immunosuppressive mediators, CAR-T cells can be co administered with matched small molecule pathway inhibitors to restore functional responsiveness. For example, inhibitors of TGF-β signalling, the IDO pathway, or adenosine signalling can be evaluated to determine whether organoid sensitivity to CAR-T mediated cytotoxicity is improved. Driven by parallel, head to head comparisons across candidate combinations, investigators can identify the most synergistic regimens and define dose or schedule dependence, including whether pharmacologic preconditioning of organoids enhances subsequent CAR-T efficacy (Song et al., 2022; Beavis et al., 2017). Beyond CAR-T potentiation, organoid systems can also profile safety liabilities, including whether combination treatment amplifies cytokine release or induces non specific damage to organoid tissue, thereby anticipating potential toxicities (Liang et al., 2024). Overall, organoids provide a controllable, higher throughput platform to optimise CAR-T based combination strategies before clinical deployment, maximising benefit while mitigating risk, and enabling more evidence grounded clinical trial design to accelerate translation of the most promising regimens to patients. Representative examples spanning target discovery and validation, safety profiling, and patient-specific response prediction using organoid–CAR-T integration are summarized in Table 3, providing a bridge to the patient-avatar applications discussed next.

**TABLE 3:** Representative examples of CAR-T and organoid integration at various research stages.

Stage	References	Model used	Main findings	Highlights	Limitations
Target Discovery and Validation	Yu et al. (2021)	Patient derived bladder cancer organoids BCOs were established and characterized, and an antibody panel was used to profile the surface antigen repertoire of multiple CAR recognizable targets	Based on the MUC1 antigen profile, researchers engineered second-generation CAR-T cells targeting MUC1 and co-cultured them with patient-derived tumor organoids. Results demonstrated significant antigen-specific cytotoxicity exclusively in MUC1-positive organoids, manifested as directed CAR-T cell migration, organoid structural disintegration, and tumor cell lysis. Functional assays further confirmed antigen-dependent increases in granzyme B and LDH release levels within the co-culture system	Patient-derived organoids retain the histological and molecular characteristics of the primary tumor, providing a practical research platform for *in vitro* personalized functional assessment of CAR-T responses	Restricted to tumor epithelial cells, lacking immune-suppressive cells and stromal vascular components, it has limited predictive capability for *in vivo* infiltration barriers and immune-suppression mechanisms
Target Discovery & Validation	Jacob et al. (2020a)	Patient derived glioblastoma organoids GBO were directly cultured from fresh surgical specimens without single cell dissociation, preserving EGFRvIII heterogeneity	EGFRvIII-specific CAR-T cells exhibit antigen-dependent infiltration, proliferation, and activation in glioblastoma organoids, inducing tumor cell apoptosis (elevated cleaved caspase 3) and effector cytokine release (IL-2, TNF-α, IFN-γ). However, residual EGFR-positive/EGFRvIII-negative cells indicate antigen escape	In a patient derived three dimensional model, infiltration, proliferation, cell death, target antigen loss, and cytokine release were quantified simultaneously, supporting evaluation of CAR-T responses against region specific or subclonal mutant targets	In a patient derived three dimensional model, infiltration, proliferation, cell death, target antigen loss, and cytokine release were quantified simultaneously, supporting evaluation of CAR-T responses against region specific or subclonal mutant targets
Safety Assessment	Harter et al. (2024)	Donor matched normal intestinal organoids and colorectal tumor organoids tumor organoids were established as paired models, and PBMCs were added for co culture to enable simultaneous readouts of tumor efficacy and normal epithelial injury	T cell bispecific antibodies targeting EpCAM or CEA induce T cell infiltration, activation, and cytokine release in normal organoid epithelium, triggering epithelial cell apoptosis; In contrast, tumor organoids exhibited greater resistance to injury, with lower CAR-T infiltration efficiency and relatively intact core structures. Toxicity intensity was significantly influenced by target accessibility, antibody affinity, and dose, and demonstrated donor-to-donor variability	Paired tumor and normal organoids in the same system enabled combined efficacy and safety assessment and were better suited to define on target off tumor risk and therapeutic windows, supporting candidate screening and parameter optimization	The research focuses on T cell bispecific antibodies rather than CAR-T cells, and extrapolation to CAR-T requires direct validation. This *in vitro* system lacks systemic metabolism, circulating immunity, and inter-organ interactions, making it difficult to predict systemic toxicities such as cytokine release syndrome. Furthermore, its limited observation window prevents assessment of long-term tissue damage and changes in regenerative capacity

## 4 Organoids as patient-specific avatars for precision CAR-T therapy

### 4.1 Functional readouts of autologous tumor organoid-autologous CAR-T co-culture

Autologous tumor organoid-autologous CAR-T co-culture provides a clinically relevant *ex vivo* assay to quantify patient-specific CAR-T function by pairing patient-derived tumor organoids with CAR-T cells generated from the same individual (Ohta et al., 2024). This matched system preserves key tumor-effector interfaces and supports integrated measurements of antitumor activity, including organoid killing and apoptosis, effector cytokine release, T-cell infiltration, and therapy-associated phenotypic remodeling (Zou et al., 2021). Antigen-dependent cytotoxicity is typically captured within short co-culture windows; for instance, increased intratumoral caspase-3 cleavage and elevated effector cytokines in supernatants are generally observed only when organoids express the cognate CAR target and are absent in appropriate controls (Martins et al., 2024).

Organoid architectures enable direct visualization of CAR-T migration and tumor entry in three dimensions using live imaging and immunofluorescence, including peripheral accumulation, progressive penetration, and formation of cytolytic contacts culminating in apoptosis or necrosis (Cho et al., 2024). Because dense extracellular matrices can impede immune-cell motility, matrix dilution, partial matrix digestion, or suspension-based formats are often adopted to improve access and enhance the interpretability of infiltration metrics (Magré et al., 2023). Recovered CAR-T cells can subsequently be profiled for activation and exhaustion by flow cytometry; organoid-derived suppressive mediators such as TGF-βand IL-10 may induce inhibitory states, allowing negative regulatory circuits to be captured *ex vivo* (Jassin et al., 2025a).

Emerging clinical concordances suggest predictive potential. In glioblastoma, patient-matched organoid co-cultures showed that organoid lysis tracked radiographic tumor reduction, while supernatant cytokine kinetics mirrored cerebrospinal fluid cytokine dynamics after infusion, characterized by an early rise followed by a decline within approximately 1 week (Logun et al., 2025). These observations indicate that co-culture readouts may provide response-associated signatures and inform inflammatory intensity in anatomically constrained sites (Ijaz et al., 2025). Key limitations include manufacturing time and tissue requirements, restricted immune complexity in many platforms that omit myeloid and other suppressive populations, and the absence of vasculature that limits modeling of trafficking and extravasation (Neal et al., 2018). Nonetheless, autologous organoid-CAR-T co-culture remains a rapid, repeatable, mechanistically informative functional assay that can support individualized cellular-therapy optimization (Thorel et al., 2024).

### 4.2 Parallel evaluation of CAR configurations and combination regimens to converge on individualized strategies

Patient-derived organoids allow parallel *ex vivo* benchmarking of multiple CAR-T constructs and a focused set of adjunct regimens from a single specimen, enabling rapid prioritization of an individualized strategy without iterative patient exposure. This is particularly valuable in solid tumors, where antigen heterogeneity and immunosuppressive niches often require tailored CAR architectures together with rational combinations to achieve clinically meaningful activity (Ning et al., 2024).

In immune-organoid co-culture, CAR performance can be compared head-to-head within the same patient-matched tumor context using readouts such as tumor killing, persistence-associated phenotypes, and early dysfunction or exhaustion signatures (Zou et al., 2021). For heterogeneous antigen landscapes, dual-target or tandem CAR designs can be directly stress-tested for their ability to mitigate antigen escape and improve completeness of tumor clearance (Zou et al., 2021; Zhu et al., 2025b). In glioma organoids, a bispecific CAR targeting EGFR and IL13Rα2 has shown superior elimination relative to single-antigen constructs, consistent with reduced single-antigen escape (Schmidts et al., 2023).

Organoid platforms also support systematic assessment of combination regimens that either relieve immune suppression or increase tumor-intrinsic vulnerability. Checkpoint blockade can be evaluated by adding PD-1 or PD-L1 pathway inhibition and quantifying whether CAR expansion and cytotoxicity improve under checkpoint relief, especially when co-culture reveals early exhaustion-associated features (Kastenschmidt et al., 2024). Similarly, co-administration of targeted agents can test whether pharmacologic sensitization enhances CAR efficacy; for example, in glioblastoma organoids, adding the IAP inhibitor birinapant to an EGFRvIII CAR-T system enhanced tumor clearance, linked to NF-κB activation and increased susceptibility to CAR-derived inflammatory cues (Song et al., 2022). TGF-β-mediated suppression can be addressed by comparing pharmacologic pathway inhibition with engineered resistance strategies within the same patient context, and cholangiocarcinoma organoid studies report improved CAR activity when inhibitory axes such as TGF-β receptor signaling, PD-1, or TIGIT are disrupted (Qiao et al., 2023).

Overall, while not intended for unrestricted high-throughput discovery, organoid co-culture is well suited to ranking a prioritized panel of CAR constructs and adjunct interventions as a miniature pre-treatment simulation, balancing tumor eradication against preservation of CAR functional state (Grönholm et al., 2021). Continued standardization and partial automation may facilitate routine organoid-guided selection of CAR designs and combination regimens with attention to both efficacy and tolerability (Liu et al., 2026; Yin et al., 2026).

### 4.3 Boundary conditions for clinically actionable translation from tolerance mechanisms

CAR-T efficacy in solid tumors is commonly constrained by three convergent programs: target antigen loss or heterogeneity, microenvironmental suppression, and progressive T-cell dysfunction with exhaustion-like features (Lindo et al., 2020). Tumor organoid co-culture offers a practical framework to identify which mechanism dominates in a given patient context and to define the conditions under which *ex vivo* findings can inform treatment decisions rather than remain descriptive (Lindo et al., 2020).

Organoid systems can operationalize antigen escape by tracking selective pressure: CAR-T-mediated depletion of antigen-positive cells followed by expansion of antigen-negative residual populations provides direct evidence for escape and rationalizes multi-target designs (Jacob et al., 2020a). They can also expose suppressive niches when impaired killing and limited CAR-T expansion coincide with elevated inhibitory mediators such as TGF-βor IL-10; causality can be stress-tested by adding pathway blockade (e.g., TGF-β or PD-1/PD-L1) and quantifying functional rescue (Ning et al., 2024). Exhaustion is partially measurable *via* prolonged or repeated stimulation, where escalating dysfunction and exhaustion-marker upregulation may be mitigated by checkpoint relief in selected settings (Cherkassky et al., 2016).

Clinical translation requires converting these mechanistic readouts into decision rules: actionable biomarkers, indication refinement, and explicit selection logic for CAR design and combination therapy (Yuki et al., 2020). Antigen heterogeneity supports eligibility thresholds for single-*versus* multi-target strategies, while high suppressive mediator signatures justify pre-specified add-on derepression approaches (Zhang et al., 2025). Functional organoid response can prioritize patients likely to benefit and flag scenarios where CAR-T should be modified or combined before exposure (Mei et al., 2024).

Implementation is bounded by speed, reproducibility, and feasibility. Assays must fit clinical decision windows, and predictions must be robust enough to justify treatment adaptation; individualized CAR redesign is further constrained by manufacturing and regulatory timelines (Shah et al., 2023). Because many organoid platforms incompletely recapitulate vascular trafficking and full stromal or immune ecology, organoid-guided choices should be triangulated with complementary models and strengthened through standardization, quality systems, and prospective validation (Yao et al., 2025).

## 5 Current limitations and future engineering directions

Although organoid models hold considerable promise for CAR-T research, several technical bottlenecks must be overcome to fully realise their utility. Sustained immune maintenance is a major challenge, because most current organoid immune cell coculture systems struggle to preserve immune cell viability and effector function over prolonged periods, and they rarely support the stable coexistence of multiple immune lineages (Wang Q. et al., 2025). Without replenishment from an *in vivo* hematopoietic system, effector T cells *in vitro* readily undergo functional attrition or apoptosis due to insufficient stimulation and metabolic support, limiting how accurately organoid platforms can model long term CAR-T persistence (Zhu W. et al., 2025).

To address this, researchers are exploring practical strategies such as periodic addition of fresh immune cells, supplementation with cytokines including interleukin 2 to sustain T cell expansion, and incorporation of supportive cells that provide antigen presentation and costimulatory signals to extend immune durability (Cattaneo et al., 2020). In parallel, immune tissue organoids, for example lymph node like organoids, are being developed and combined with tumour organoids to approximate a more complete immune circuit that can support prolonged CAR-T function (Votanopoulos et al., 2020).

Matrix standardisation remains a central bottleneck in organoid culture, which still largely depends on animal derived matrices such as Matrigel whose composition is complex and whose mechanical and biochemical properties vary substantially across lots (Aisenbrey and Murphy, 2020). Such lot to lot variability can lower culture success rates and, more importantly, compromise the reproducibility of experimental readouts (Aisenbrey and Murphy, 2020). By driving a shift toward defined microenvironments through tunable extracellular matrix mimicry while eliminating the uncertainty of animal components, synthetic alternatives such as peptide based hydrogels and gelatin methacryloyl are being actively developed (Balion et al., 2020). As more synthetic matrices become commercially available and are rigorously validated for equivalence to conventional substrates, organoid studies should be performed under more uniform conditions, thereby improving cross laboratory comparability. Standardisation is also required for other steps, including media formulations and dissociation and passaging procedures, and international collaborative networks have begun to share detailed protocols and quality control standards to facilitate the emergence of harmonised field wide practices (Tiriac et al., 2018). This harmonisation is also pivotal for regulatory acceptance of organoid models as tools for drug evaluation.

Organoid-CAR-T co-culture generates high-dimensional datasets spanning transcriptomes, secretomes, and longitudinal imaging; converting these measurements into actionable insights for CAR-T development remains a major challenge. AI, especially machine-learning methods, can interrogate large-scale multi-omics to identify the key features and interaction patterns that shape organoid-CAR-T crosstalk (Ha et al., 2023; Balkhair and Albalushi, 2025; Ma et al., 2025). For example, AI can uncover organoid gene-expression signatures that predict strong CAR-T activity, or cytokine-trajectory patterns that consistently precede CAR-T exhaustion. These signals can sharpen in vitro-in vivo efficacy linkage models, thereby guiding CAR-T product optimization and patient selection. Multi-omics integration can also improve the organoid model itself: by comparing molecular states across culture conditions, AI can prioritize the variables that most strongly drive phenotypic outputs and feed this information back into process refinement (Bai et al., 2024). Looking forward, organoid platforms are likely to merge with AI-native analytics to enable automated, intelligent experimentation, where robotic high-throughput execution paired with real-time AI monitoring reduces operator-dependent error and improves inter-batch reproducibility.

Achieving comprehensive deployment of organoids in CAR-T development hinges on resolving practical constraints, particularly scalable high-throughput platforms and clinically actionable translation (Ma et al., 2025; Bose et al., 2021). To fully leverage organoid models during screening and optimization, experimental throughput must be substantially increased (Schuster et al., 2020). At present, organoid culture and readout remain largely manual, time intensive, and labor demanding; throughput scaling is therefore being driven by microfluidic organoid chips and arrayed seeding approaches that miniaturize culture into multiwell plates and chip formats, enabling parallel processing of hundreds to thousands of samples. Integration of automated liquid handling with high content imaging and computational phenotyping is accelerating the feasibility of organoid based high-throughput screening (Avci et al., 2025). For example, a patient derived tumor organoid biobank can be established to test a given CAR-T product across dozens of tumor backgrounds within a single experimental campaign, rapidly delineating its indication spectrum and resistance liabilities.Clinical translation and the maturation of a regulatory framework are equally consequential (Zhou et al., 2025). At present, organoids are primarily research tools, and their role in clinical decision making remains exploratory; regulators are still developing guidance on how organoid derived evidence should be generated, validated, and interpreted. Prospective studies that demonstrate a robust, reproducible correlation between organoid responses and patient outcomes will be essential before organoid testing can be recognized as a companion diagnostic modality.

Accordingly, for each newly developed CAR-T product, it will be necessary to specify the performance thresholds, including the sensitivity and specificity required for predicting clinical efficacy, and to define how such an *ex vivo* assay should be classified and governed within regulatory pathways (Xiang et al., 2024). As standards and guidance converge, organoid platforms are likely to be incorporated into CAR-T development and approval as a formal component supporting efficacy evidence and safety surveillance.In summary, addressing bottlenecks in immune maintenance, extracellular matrix standardization, data integration, and throughput, while advancing industry standards and regulatory acceptance, is pivotal for fully embedding organoid technology into CAR-T R and D and ultimately delivering patient benefit. With these barriers progressively removed, organoid platforms may become a central engine for CAR-T innovation, enabling immunotherapies that are more efficient, more precise, and safer.

## 6 Conclusion

Organoid enabled tumor recapitulation is advancing CAR-T cell therapy toward higher precision and efficiency. By reconstructing clinically relevant tumor complexity *ex vivo*, organoid platforms narrow the translational gap between animal models and human disease and provide a scalable, human anchored testbed for CAR-T development. From antigen prioritization and functional validation to patient specific response prediction and safety de risking, organoids deliver actionable readouts across the full research pipeline. This organoid guided iteration drives faster identification of tractable targets, more rational optimization of CAR design, and earlier recognition of resistance mechanisms and clinically relevant toxicity liabilities, including on target off tumor effects and inflammatory toxicities. Collectively, these capabilities can shorten development cycles, reduce late stage clinical attrition, and ultimately deliver safer, more effective therapies for patients.

Organoid models still face constraints in preserving immune diversity, achieving standardisation, and enabling scalable deployment, but these limitations are steadily being reduced through technological advances and multidisciplinary integration. Driven by the incorporation of artificial intelligence, multiomics analytics, and organ on a chip systems, organoid platforms are expected to gain further predictive power and clinical relevance. In the near future, organoids will likely evolve from versatile laboratory tools into an indispensable cornerstone of CAR-T therapy development. With rigorous validation in organoid systems, more intelligently engineered CAR-T cells can be designed and refined before clinical entry to match tumour types and patient specific features. Looking ahead, organoid technology, together with CAR-T cell engineering, genome editing, and immunomodulatory strategies, may help usher in a new era of cancer immunotherapy. By improving both efficacy and safety, organoid enabled CAR-T approaches could benefit a broader population of patients and bring precision medicine closer to routine practice.
